# Effects of *Lactobacillus plantarum* PS128 on Children with Autism Spectrum Disorder in Taiwan: A Randomized, Double-Blind, Placebo-Controlled Trial

**DOI:** 10.3390/nu11040820

**Published:** 2019-04-11

**Authors:** Yen-Wenn Liu, Min Tze Liong, Yu-Chu Ella Chung, Hui-Yi Huang, Wu-Shun Peng, Yun-Fang Cheng, Yu-Siou Lin, Yu-Yu Wu, Ying-Chieh Tsai

**Affiliations:** 1 Institute of Biochemistry and Molecular Biology, National Yang-Ming University, Taipei 11221, Taiwan; skywenn@gmail.com (Y.-W.L.); catpet1022@hotmail.com (W.-S.P.); hiyunfang@gmail.com (Y.-F.C.); 2 Microbiome Research Center, National Yang-Ming University, Taipei 11221, Taiwan; 3 School of Industrial Technology, Universiti Sains Malaysia, Penang 11800, Malaysia; mintze.liong@usm.my; 4 Institute of Epidemiology and Preventive Medicine, College of Public Health, National Taiwan University, Taipei 10050, Taiwan; eesabella1126@gmail.com; 5 Department of Psychology, National Taiwan University, Taipei 10090, Taiwan; huanghuyiii@gmail.com; 6 Department of Psychology, National Chengchi University, Taipei 11605, Taiwan; linyusiou@gmail.com; 7 YuNing Clinic, Taipei 10664, Taiwan

**Keywords:** autism spectrum disorder (ASD), psychobiotic, *Lactobacillus plantarum* PS128, hyperactivity, anxiety

## Abstract

This four-week, randomized, double-blind, placebo-controlled study investigated the effects of *Lactobacillus plantarum* PS128 (PS128) on boys with autism spectrum disorder (ASD) aged 7–15 in Taiwan. All subjects fulfilled the criteria for ASD diagnosis of DSM-V and the Autism Diagnostic Interview-Revised (ADI-R). Questionnaires used for the primary outcome measure include the Autism Behavior Checklist-Taiwan version (ABC-T), the Social Responsiveness Scale (SRS) and the Child Behavior Checklist (CBCL). The Swanson, Nolan, and Pelham-IV-Taiwan version (SNAP-IV) and the Clinical Global Impression-improvement (CGI-I) were used for the secondary outcome measure. The results showed that PS128 ameliorated opposition/defiance behaviors, and that the total score of SNAP-IV for younger children (aged 7−12) improved significantly compared with the placebo group. Additionally, several elements were also notably improved in the PS128 group after 28-day consumption of PS128. Further studies are needed to better clarify the effects of PS128 for younger children with ASD on broader symptoms.

## 1. Introduction

Autism spectrum disorder (ASD) is a specific neurodevelopmental condition characterized by core symptoms, persistent deficits in social communication and social interactions across multiple contexts, accompanied by restricted and repetitive patterns of behaviors, interests, and/or activities [1]. ASD is a complex disorder affecting many facets of life. While its exact underlying causes remain unknown, ASD has been hypothesized to result from a combination of various factors ranging from genetics [2,3] to environmental factors [4,5,6]. According to the Centers for Disease Control and Prevention, the prevalence of ASD in the USA has increased from 4.5 in 10,000 children in 1966 [7,8] to 1 in 110 in 2006 [9] and 1 in 59 children aged 8 years in 2014 [10], respectively, mainly due to increased awareness and better diagnostic methods. In Taiwan, more than 13,000 people have been diagnosed with ASD according to the data from the Ministry of Health and Welfare, Taiwan in 2018. Furthermore, the male patients are seven-fold as many as the female ones (see the supplementary Figure S1).

In addition to the aforementioned core symptoms, attention deficits are commonly associated with ASD as more than half of the individuals with ASD also meet the diagnostic criteria for attention deficit hyperactivity disorder (ADHD) [11,12]. In children with ADHD, social communication deficit was also highly common [13]. Furthermore, children exhibiting ADHD-like behaviors in Taiwanese families are often mistakenly assumed to be oppositional and defiant instead, i.e., oppositional defiant disorder (ODD) [14].

Two antipsychotics, risperidone and aripiprazole, were approved by the U.S. FDA to treat irritability associated with autistic disorders for patients aged 5–16 and 6–17, respectively [15,16]. However, the FDA did not yet approve the drugs for the indications associated with the core symptoms of ASD. The mainstream interventions for alleviating ASD symptoms involve psychosocial strategies, such as educational interventions, speech therapies, behavioral interventions, developmental therapies and parenting skill training programs, with varying effects on improving communication and social behaviors [17,18]. Dietary and nutritional supplements, such as ketogenic diet [19,20], vitamins [21], fatty acids [22,23,24], and gluten-free [19], were reportedly to have certain effects on various ASD symptoms. However, no sufficient evidence found was adequate to support the efficacy on ASD [25,26,27].

The gut-brain axis is a bidirectional, neurohumoral communication system that ultimately affects the homeostasis of the gastrointestinal, immune, and neural systems [28,29]. More than 10^13^–10^14^ bacteria weighing over 1 kg are present in the human gut, creating a microecology and reservoir for various metabolites that link intestinal functions with the emotional and cognitive functions of the brain. Based on the accumulative evidence, the modulation of gut-brain axis functions improves stress responses and overall behaviors in both animals [30,31,32] and humans [33,34]. People with ASD showed a high frequency of gastrointestinal problems [35,36,37]. Furthermore, the altered gut microbiota composition in ASD subjects was reported in different studies [38,39,40,41]. Kang et al. reported results of an open-label trial using microbiota transfer therapy for autism and gastrointestinal symptoms (NCT02504554) [42]. Eighteen autism subjects (aged 7–16) received intervention with microbiota transfer therapy and improvement of autism symptoms, assessed by Childhood Autism Rating Scale (CARS), Aberrant Behavior Checklist and Social Responsiveness Scale (SRS), and gastrointestinal symptoms, assessed by Gastrointestinal Symptom Rating Scale (GSRS), were reported in this study [42]. The above results suggested that targeting gut may bring beneficial impact on people with ASD.

Probiotics are “live microorganisms which when administered in adequate amounts confer a health benefit on the host” [43]. *Lactobacillus*, one of the most commonly administered probiotic genera, has a long history of safe use and varying benefits to hosts, ranging from gastrointestinal health to vitamin synthesis and the alleviation of metabolic disorders [44,45]. A new class of probiotics known as “psychobiotics” is defined as “a live organism that, when ingested in adequate amounts, produces a health benefit in patients suffering from psychiatric illness” [46]. By conducting behavioral tests in animal models, psychobiotic strains were reported to improve depression-like behaviors [47,48,49], anxiety-like behaviors [30,47,48,49,50,51,52], memory [53,54], cognition [55], and autism-like behaviors [56]. To date, the only bacteria reported to improve autism-like behaviors in a maternal immune activation mouse model is *Bacteroides fragilis* (ATCC 9343) [56]. *Lactobacillus plantarum* PS128 (PS128) was isolated from fu-tsai, which is a spontaneously fermented mustard product in Taiwan and is generally used as a food ingredient [57]. We previously reported the beneficial effects of PS128 as a psychobiotic strain. In the maternal separation-induced early life stress in C57BL/6 male mice model, oral administration of PS128 significantly reduced the levels of corticosterone and IL-6 in serum and increased the dopamine level in the prefrontal cortex. Behavioral assessments using the open field test and elevated plus maze revealed that PS128 reduced anxiety in early life stress mice [48] while improving locomotor activities and anxiety-like behaviors, accompanied by increased levels of both serotonin and dopamine in the striatum of germ-free mice [51]. 

According to various animal studies, PS128 can modulate the levels of neurotransmitters in the brain [48,51]. Additionally, disturbance of neurotransmitters was reported in ASD patients [58,59,60], so we hypothesized that PS128 may have beneficial effects on ASD symptoms. In this study, we utilized several validated assessment tools to evaluate the effects of PS128 on ASD symptoms.

## 2. Materials and Methods 

### 2.1. PS128 and Placebo Products

*Lactobacillus plantarum* PS128 was isolated [57], and deposited under DSMZ Accession No. DSM 28632. The genome architecture of PS128 was reported previously [61]. In the present study, the PS128 product was provided by Bened Biomedical Co., Ltd. in the final form of capsules containing creamy white powders. The probiotic capsules weighed 425 ± 25 mg and contained 3 × 10^10^ CFU/capsule of PS128 with microcrystalline cellulose as the carrier, whereas the placebo capsules only contained microcrystalline cellulose. All capsules were identical in taste and appearance and were stored at a refrigerated temperature (4–8 °C).

### 2.2. Study Design

#### 2.2.1. Selection of Subjects

Subjects were screened based on inclusion and exclusion criteria. The inclusion criteria were boys aged 7 to 15 years diagnosed with ASD based on the Diagnostic and Statistical Manual of Mental Disorders, 5th edition (DSM-V) criteria [1]: Major caregiver was asked to provide the physical and mental disability card provided by the Taiwan government and the researcher of this study check the ICD-9 to be 299.00. Considering the prevalence of ASD is about four times more common among boys than among girls [10], we only included boys with ASD into this study. The exclusion criteria included the consumption of prescribed antibiotics and yogurt or probiotic products two weeks prior to enrollment. Participants were allowed to continue their regular medications, treatment and therapies, with the exception of antibiotics, and were asked to refrain from consuming yogurt or probiotic products during the study period. Written informed consent was obtained from all subjects and the parents or caregivers of subjects prior to the start of the study.

#### 2.2.2. Study Protocol

This study had a double-blind, randomized, parallel, placebo-controlled design and planned to include 80 subjects. Because this is the first study to investigate the effects of PS128 on children with ASD, the sample size was not calculated based on the improvement of outcome measured, effect size, or errors for type I and type II. Randomization was performed upon confirmation of the inclusion and exclusion criteria. Eligible subjects were randomly allocated into the two arms of the study in a 1:1 ratio, according to randomly permuted blocks within the strata of two assignments, the probiotic group (PS128) and placebo group, using treatment codes. Randomization was performed by a research assistant who had no contact with the participants. The allocation sequence was not available to any member of the research team, the physician or the participants. This study was conducted according to the guidelines described in the Declaration of Helsinki. All procedures were conducted by trained testers at the YuNing Psychiatry Clinic, with approval by the Institutional Review Board of Antai Medical Care Cooperation, Antai Tian-Sheng Memorial Hospital (TSMH IRB No./Protocol No: 15-075-A2). The study protocol was registered on the Australian New Zealand Clinical Trials Registry (ANZCTR; Trial ID: ACTRN12616001002471). The primary outcomes of this study were changes in the Autism Behavior Checklist-Taiwan version (ABC-T) questionnaire, the Social Responsiveness Scale (SRS) scores, and the Child Behavior Checklist (CBCL) questionnaire, and the secondary outcomes were improvement in the Chinese version of the Swanson, Nolan, and Pelham-IV (SNAP-IV) assessment and the Clinical Global Impression-Improvement (CGI-I). During participation in this trial, all subjects were further confirmed the diagnosis of ASD with the Autism Diagnostic Interview-Revised (ADI-R) [62] performed by trained testers.

### 2.3. Analyses

The Clinical Global Impression-Severity (CGI-S) and Clinical Global Impression-Improvement (CGI-I) forms were completed by Dr. Yu-Yu Wu at baseline (week 0) and upon completion of the intervention (week 4), respectively. Parents or caregiver completed subsequent scores for the ABC-T, CBCL, SRS, and SNAP-IV questionnaires. This study comprised two visits, where the baseline visit involved enrollment, randomization prior to the start of the study, and assessments using questionnaires, whereas the second visit involved a review of the subject’s medical history and reports of adverse events (week 4).

#### 2.3.1. ABC-T

The ABC-T is a 47-item questionnaire used to assess behavioral problems in children with intellectual and developmental disabilities which was modified from Autism Behavior Checklist of Autism Screening Instrument for Education Planning-Third edition [63] by Dr. Yu-Yu Wu and colleagues. This validated tool is divided into five subscales, including problems related to sensory (sensation and perception; eight items), relating (relation and connection; 11 items), body and object use (physical activity and rigid use of objects; 12 items), language (communication and interaction; eight items) and social and self-help (adaptability and self-care; eight items). ABC-T items were rated as “yes” (rated as 1, with symptom) or “no” (rated as 0, without symptom) for each question during the assessment.

#### 2.3.2. SRS-Taiwan Version

The SRS is a validated 65-item assessment tool designing to assess social communication and interactions, as well as restricted interests and repetitive behaviors [64]. These assessments are categorized into social awareness, social communication, social emotion and autistic mannerisms [65]. 

#### 2.3.3. CBCL

The CBCL L is a 113-item questionnaire which assesses eight empirically based syndrome scales, including aggressive behaviors, anxiousness, attention problems, rule-breaking behavior, somatic complaints, social problems, thought problems and withdrawal issues [66,67,68].

#### 2.3.4. CGI-S and CGI-I

The CGI-S and CGI-I questionnaires both comprise items measured on a seven-point scale that are rated by a clinician to determine symptom severity and improvement, respectively [69].

#### 2.3.5. SNAP–IV

SNAP-IV was used to evaluate the ADHD and ODD in children aged 6–15. It is useful for interpretation of the effects of interventions on patients enrolled in clinical studies [70]. The Taiwan version of the SNAP-IV consisted of 26 items reflecting DSM-IV symptoms of attention-deficit/hyperactivity disorder (ADHD) (18 items) and oppositional and defiance problems (8 items) [14,70,71,72]. 

### 2.4. Statistical Analyses

Data were analyzed using GraphPad Prism (Version 7; GraphPad Software, San Diego, CA, USA) and Excel. We conducted two-tailed t-test to compare demographics (parametric test), clinical characteristics (ADI-R scores, CGI-S, and CGI-I) and scores of outcomes measurement between PS128 group and placebo group at baseline and week 4. For the exploratory analysis stratified by age, we divided the subjects into the age groups of 7–12 and 13–15 years. We further applied independent *t*-test to compare the differences of questionnaires (week 4 score—baseline score) between the PS128 and placebo groups. Scores of baseline and week 4 were analyzed by using a paired *t*-test for within group analysis. A two-tailed significance level of *P* < 0.05 was considered statistically significant.

## 3. Results

### 3.1. Baseline Demographics

**Figure 1:**
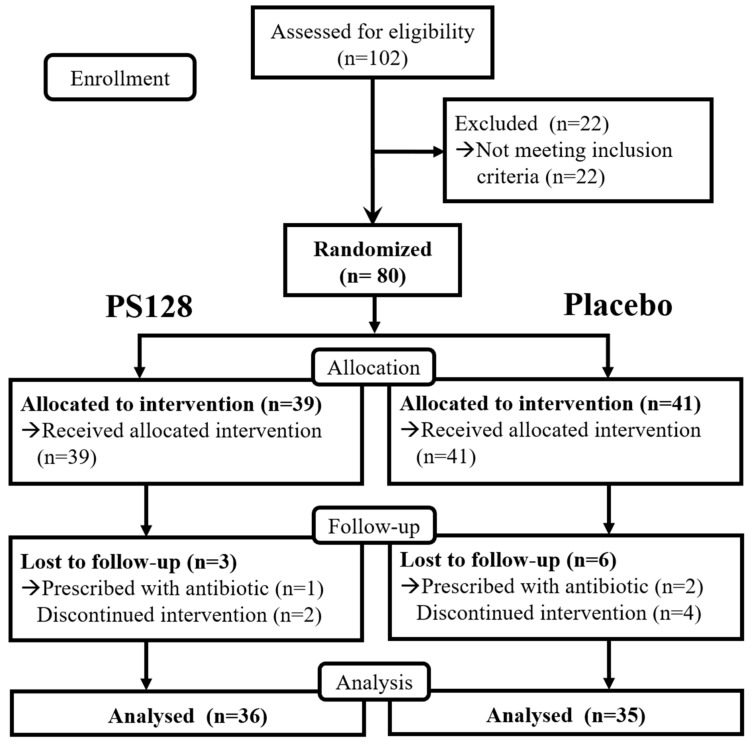
Trial profile.

Between 24 February 2016, and 25 August 2016, we randomly assigned 80 subjects to treatment: Thirty-nine assigned to PS128 and 41 assigned to placebo. Of the eligible 80 subjects aged between 7 and 15 years, three and six subjects dropped out within the PS128 and placebo groups, respectively, yielding 71 subjects at the end of the study period (PS128, *n* = 36; placebo, *n* = 35; Figure 1). One subject in the PS128 group and two subjects in the placebo group dropped out because of prescribed antibiotics. One subject in the PS128 group and four subjects in the placebo group withdrew informed consent. One subject in the PS128 group was discontinued participating because not completed the ADI-R. No adverse event was reported in this study. No subjects or their parents reported any gastrointestinal intolerance or allergic responses during their participation. Insignificant differences in the general characteristics of age, height and weight were observed between the PS128 and placebo subjects at baseline (week 0) (*P* > 0.05; Table 1). Subjects were confirmed for the diagnosis of ASD by ADI-R assessment by experienced researchers. Subjects from both groups fulfilled the criteria for autism, with scores exceeding the cut-off values for all four domains, reciprocal social interaction, language and communication (verbal and non-verbal), stereotyped repetitive behaviors or interests, and age-of-onset, and scored similarly (*P* > 0.05; Table 1). The CGI-S scores of the PS128 and placebo groups were also similar at baseline (*P* = 0.26) (Table 1). This section may be divided by subheadings. It should provide a concise and precise description of the experimental results, their interpretation as well as the experimental conclusions that can be drawn.

### 3.2. Outcomes Measurement

Analysis of all outcomes was assessed between PS128 and placebo groups. Further age stratification was conducted to analyze the outcomes between groups. An exploratory analysis was carried on for baseline and endpoint within the group.

#### 3.2.1. CGI-I

The CGI-I scores for both groups were equivalent to “minimally improved” (3.64 and 3.66 for the PS128 and placebo groups, respectively; *P* = 0.94) (Table 2).

#### 3.2.2. ABC-T

There was no difference between the placebo and the PS128 groups in the total ABC-T-score and subscale scores both on baseline and week 4 (Table 2). Further exploratory analysis revealed that four-week consumption of PS128 showed a trend to reduce the scores for body and object use (*P* = 0.04; Table 3).

#### 3.2.3. SRS

The total score and subscale scores of SRS between PS128 and placebo groups were similar both on baseline and week 4 (Table 2). Further exploratory analysis showed nominal reduction of SRS-total score of PS128 group between baseline and week 4 (*P* = 0.04), however, the children in the placebo control group did not show improvements over time (Table 3).

#### 3.2.4. CBCL

There was no difference between placebo and treated groups in the total CBCL scores both on baseline and week 4 (Table 2). However, results of exploratory analysis indicated that four-week consumption of PS128 nominally reduced the scores for anxiety (*P* = 0.02), and rule-breaking behaviors (*P* = 0.02) (Table 3). Children in the placebo group showed a reduction in scores for problems related to externalization (*P* = 0.02) over four weeks, but children consuming PS128 did not show this trend (Table 3).

#### 3.2.5. SNAP-IV

The scores of SNAP-IV were similar between the PS128 and placebo groups both on baseline and week 4 (Table 2). Exploratory analyses showed that reduced total scores (*P* = 0.01), hyperactivity and impulsivity (*P* = 0.04), and opposition and defiance (ODD; *P* = 0.045) over four weeks were observed in the PS128 group; the placebo group did not exhibit these changes (Table 3).

### 3.3. Analysis Stratified by Age

Further stratified analysis by age (7–12 years old, elementary school; 13–15 years old, junior high school) was carried on for all outcomes measured. In the elementary school subjects, the scores of social awareness of the placebo group were lower than the PS128 group at both baseline (*P* = 0.02) and at week 4 (*P* = 0.04) (Table 4). By comparing the changes over time (week 4 score—baseline score), the PS128 group showed improved opposition/defiance (*P* = 0.03) and total score (*P* = 0.02) of SNAP-IV compared with placebo group (Table 4). The exploratory analysis within groups showed that CBCL-anxiety (*P* = 0.01), CBCL-rule-breaking behaviors (*P* = 0.01), SNAP-IV-inattention (*P* = 0.03), SNAP-IV-hyperactivity/impulsivity (*P* = 0.02), SNAP-IV-Opposition/defiance (*P* = 0.02), and total score (*P* = 0.004) were nominally reduced in children aged 7–12 years old in PS128 group over the four-week intervention. In the placebo group, younger children showed slight improvement in CBCL-external (*P* = 0.04) (Table 3).

## 4. Discussion

This study aimed to study the effects of psychobiotic PS128, a specific probiotic strain, on behaviors of boys with ASD, aged 7–15 in Taiwan. Randomization was performed, and the general characteristics of the children who were administered PS128 or placebo at baseline and after four weeks were similar (Table 1). No adverse event was reported. CGI-S and CGI-I were assessed by the Principle Investigator of this study, Dr. Wu, at baseline and day 28, respectively. In this study, subjects in both the PS128 and placebo groups showed similar severity at baseline and improvements at week 4, according to CGI-S and CGI-I scores, suggesting that the autism traits did not change between the two visits. However, subjects in the PS128 group showed nominal improvement in several elements, including ABC-T-body and object use, SRS-total score, CBCL-anxiety, CBCL-rule breaking behavior, SNAP-IV-hyperactivity/impulsivity, SNAP-IV-opposition/defiance, and SNAP-IV-total score. Moreover, younger subjects (elementary school, aged 7–12) in the PS128 group showed improvement in SNAP-IV-opposition/defiance and SNAP-IV-total score, while the counterpart in the placebo group did not.

Although PS128 did not exert substantial effects on most of the subscales of the ABC-T, an improvement was observed over time in problems related to body and object use, a common spectrum of rigid behavioral problems exhibited by children with ASD (*P* = 0.04; Table 3). The SRS scores are continuously distributed, reproducible, unrelated to intelligence level or age, and highly valid [73,74]. In this study, the total score of SRS was nominally decreased in the PS128 group after intervention, suggesting that PS128 may improve behavioral or social communicative traits of children with ASD (*P* = 0.04; Table 2). The CBCL is highly relevant for distinguishing thought problems in school-age children with autism, displaying nearly 100% accuracy [75]. Based on our data, the total scores of CBCL were not significantly different between those two groups after intervention and between the two time points of each group. However, the scores associated with anxiety and rule-breaking behaviors were nominally reduced in the PS128 group after four-week consumption of PS128 (Table 2 and Table 3). These behaviors may reflect a broad class of behavioral problems commonly identified in children with autism which are often “silent” and not manifested externally, leading to potential problems with attention, defiance and self-inflicted harm. SNAP-IV was used in clinical practice for ADHD and ODD diagnosis based on the DSM-IV criteria [14]. Although the symptoms of ADHD and ODD are not the core symptoms of ASD, co-occurrence with ADHD is believed to be associated with school dysfunction for ASD children [76]. The observation from assessing SNAP-IV in the current study showed that PS128 may be beneficial for improving the ADHD-related symptoms, hyperactivity/impulsivity (*P* = 0.04), and contributed to the reduced total score (*P* = 0.02) for children with ASD (Table 2 and Table 3).

The age-stratified analysis of the results obtained from this study showed that younger subjects in the PS128 group aged 7–12, effectively elementary school age, experienced greater benefit than the older children in the junior high school age (aged 13–15; Table 3 and Table 4). The SNAP-IV-opposition/defiance was improved in the subjects aged 7–12 in the PS128 group compared with the placebo group (*P* = 0.03) and contributed to the improved SNAP-IV total score (*P* = 0.02) (Table 4). Autism therapies have been reportedly to produce the greatest gain in improving cognitive and linguistic abilities and adaptive behaviors in children with autism as young as two years old [77], which stresses the importance of early diagnosis and interventions. Although ASD can be reliably diagnosed at as early as 24 months of age, yet most children are not diagnosed until they reach the school age [78]. Based on the observation from this study, PS128 may benefit children with autism, with potentially greater benefits upon early intervention.

ASD is a broad-spectrum disorder that covers various aspects of a person’s development, as illustrated prevalently in behavioral symptoms and functions. In any typical assessments involving psychological parameters, multiple assessment tools are needed for robust diagnosis and measurement of putative treatments and efficacy [79]. Previous studies that examined the effects of interventions with probiotics on ASD were either not randomized [80], relatively small in scale, or not using ASD-specific diagnostic tools for subject recruitment [81]. To the best of our knowledge, this study represents the first trial that uses probiotics in a randomized, double-blind and placebo-controlled settings, utilizes multiple validated assessment tools specific to ASD behavioral symptoms and functions. The ABC-T specifically determines ASD symptoms [63], whereas the SRS [65], SNAP-IV [82,83] and CBCL [84] specifically evaluate behavioral symptoms that are strongly correlated with ASD. This study also utilized a validated diagnostic tool for ASD during the subject recruitment process, where all subjects were confirmed to have ASD using ADI-R [85].

There are some limitations of this study and more improvement to be expected in future studies. First of all, the duration of the intervention was only four weeks in this study and can be too short. Four-week intervention is selected conventionally in clinical trials using probiotics on ASD [86]. Shaaban et al. reported that the probiotic mixture intervention for three months significantly improved scores of Autism Treatment Evaluation Checklist (ATEC) and six-item Gastrointestinal Severity Index (6-GSI) in subjects with autism [80]. As ASD is a neurodevelopmental disorder with a wide range of symptoms and that both genetic and environmental factors are involved in the pathogenesis, a longer intervention and study duration may yield a better understanding of the effects. In addition, unlike drugs probiotics may colonize the gut [87,88], and thus a prolonged positive effect may be feasible. This study, however, did not evaluate prolonged outcomes, which may include the continuation of benefits and/or possible recurrences of disorders upon termination of consumption. Moreover, although double-blinding often adds strength to study design, this study illustrated a high placebo effect. In our study, all subjects were allowed to keep their original medication and maintain all their therapies. Therefore, the changes in outcomes measured may be attributed to either the PS128 interventions or placebo. King et al., summarized and reported that the responder rate in the placebo group ranged from 10–50% in autism trials [89]. Jones and colleagues used questionnaires scored by caregivers of subjects with autism for behavioral changes in overtime evaluation without intervention. According to their results of ABC and SRS, the response rate was 29% and 7%, respectively [90]. Our observations are similar to the aforementioned studies such that the scores of all the outcomes measured, except social awareness of SRS, decreased in the placebo group at week 4, suggesting that the placebo effect may exist in this study. It was difficult to clarify the confounding factors in this study because a complete medical and psychological treatment of the subjects was not recorded. Although reports or questionnaires performed by the caregivers are generally used for outcome measurement in psychiatric and pediatric studies, objective measurements, such as biochemical analysis may help to verify the existence of placebo effects. Furthermore, the statistic power is low. In the current study, the statistic power of all measurements ranged from 0.055–0.439 which are less than generally expected power of 0.8 [91]. If multiple-testing correction is taken into account, some observation may become not indicative. The missing data may lead to low statistic power in this study.

## 5. Conclusions

As ASD covers a broad spectrum of communicative and behavioral problems and varies between individuals significantly, it makes a single, all-effective therapy nearly impossible. Various therapies have been developed to tackle ASD on a case by case basis. Based on our data, *Lactobacillus plantarum* PS128, a reported psychobiotic, can ameliorate some autism symptoms, primarily those associated with disruptive and rule breaking behaviors and hyperactivity/impulsivity. Additionally, the efficacy of PS128 intervention seemed to be age-dependent, with better effects noticed on younger children than older children, underscoring the importance of early interventions. Taken together, it seems that psychobiotic PS128 may be beneficial for children with ASD, and that further studies remain to be done.

## Figures and Tables

**Table 1:** Demographic and clinical characteristics of the subjects at baseline.

Characteristic	PS128	Placebo	Total	*P*-Value
Sample size (*n*)	36	35	71	
Age	10.11 (2.34)	9.91 (2.33)	10.01 (2.32)	0.72
Height	144.16 (16.31)	140.63 (15.44)	142.39 (15.85)	0.38
Weight	37.31 (13.56)	35.65 (15.12)	36.49 (14.26)	0.65
CGI-S	4.86 (1.25)	5.17 (1.04)	5.01 (1.15)	0.26
**Autism Diagnostic Interview-Revised (ADI-R) Scores**				
Qualitative abnormalities in reciprocal social interaction	22.81 (4.78)	24.03 (6.55)	23.41 (5.71)	0.37
Qualitative abnormalities in communication (Verbal)	16.22 (4.66)	16.20 (4.90)	16.21 (4.75)	0.98
Qualitative abnormalities in communication (Non-verbal)	9.19 (3.18)	9.20 (2.92)	9.20 (3.03)	0.99
Restricted, repetitive, and stereotyped patterns of behavior	7.11 (2.41)	8.14 (2.48)	7.62 (2.48)	0.08
Abnormality of development evident at or before 36 months	3.86 (1.38)	3.83 (1.49)	3.85 (1.42)	0.92

The results are expressed as means (SD).

**Table 2:** Analysis of clinical outcomes.

	Baseline			Week 4		
	PS128	Placebo	P-Value	PS128	Placebo	P-Value
**CGI-I**				3.64 (1.1)	3.66 (1.00)	0.94
**ABC-T**						
Sensory	2.24 (1.41)	3.03 (1.98)	0.07	2.21 (1.58)	2.75 (1.74)	0.19
Relating	3.88 (2.77)	3.74 (2.43)	0.82	4.03 (3.08)	3.63 (2.74)	0.59
Body and object use	3.42 (2.62)	3.71 (2.71)	0.66	3.12 (2.59)	3.45 (2.68)	0.62
Language	2.47 (2.15)	3.0 (1.77)	0.27	2.15 (1.89)	2.81 (1.84)	0.16
Social and self help	3.24 (2.02)	3.59 (2.11)	0.48	3.15 (1.98)	3.52 (2.45)	0.52
Total score	15.81 (8.39)	17 (9.31)	0.59	14.67 (8.97)	16.21 (10.11)	0.53
**SRS**						
Social communication	64.06 (14.84)	64.0 (15.87)	0.99	62.17 (12.9)	63.34 (16.21)	0.75
Autism mannerisms	30.82 (6.26)	30.39 (6.58)	0.79	29.97 (7.25)	29.71 (6.53)	0.88
Social awareness	23.29 (4.22)	21.11 (5.27)	0.06	23.21 (4.89)	21.57 (5.25)	0.21
Social emotion	19.74 (4.54)	19.11 (4.34)	0.56	19.24 (4.55)	18.81 (5.14)	0.72
Total score	138.87 (24.19)	135.88 (26.04)	0.64	132.77 (22.99)	135.79 (25.79)	0.63
**CBCL**						
Anxiety	6.76 (4.85)	6.0 (4.46)	0.51	5.63 (4.34)	5.84 (4.41)	0.84
Withdrawn	4.41 (2.83)	4.68 (3.05)	0.71	4.22 (2.67)	4.50 (3.16)	0.7
Somatic complaints	2.38 (1.79)	3.13 (2.92)	0.22	2.5 (2.53)	2.41 (2.72)	0.9
Internalization	13.32 (7.98)	13.60 (8.46)	0.9	12.34 (6.83)	12.72 (8.53)	0.85
Social problems	7.65 (3.98)	7.71 (3.13)	0.95	7.38 (4.04)	7.65 (3.67)	0.78
Thoughts problems	5.73 (4.04)	6.90 (5.57)	0.35	4.84 (3.96)	6.50 (5.28)	0.17
Attention problems	10.61 (3.74)	11.12 (3.92)	0.59	10.87 (4.42)	10.90 (4.28)	0.98
Rule-breaking behavior	3.5 (3.3)	3.84 (2.74)	0.65	3.06 (3.62)	3.31 (3.15)	0.77
Aggressive behavior	8.27 (7.0)	8.71 (6.21)	0.8	8.06 (7.02)	7.94 (6.02)	0.94
External	11.73 (9.73)	12.75 (8.64)	0.67	11.1 (10.2)	11.25 (8.47)	0.95
Total score	49.63 (25.4)	50.60 (25.91)	0.89	44.34 (23.25)	49.20 (24.46)	0.53
**SNAP-IV**						
Inattention	15.18 (5.83)	15.79 (5.16)	0.66	14.39 (5.91)	15.35 (5.48)	0.5
Hyperactivity/impulsivity	10.3 (5.51)	10.97 (6.0)	0.64	9.65 (5.23)	10.25 (6.42)	0.68
Opposition/defiance	8.93 (6.09)	7.5 (5.41)	0.32	7.73 (5.04)	7.41 (5.43)	0.8
Total score	34.03 (14.61)	34.48 (13.39)	0.9	31.87 (14.26)	33.16 (15.58)	0.73

The results are expressed as means (SD). ABC-T: Aberrant Behavior Checklist-Taiwan version; CBCL: Child Behavior Checklist; CGI-I: Clinical Global Impression-Improvement; CGI-S: Clinical Global Impression-Severity; SNAP-IV: Swanson, Nolan and Pelham (SNAP)-IV-Taiwan version; SRS: Social Responsiveness Scale.

**Table 3:** Analysis of difference between baseline and week 4 of PS128 and placebo group.

	PS128			Placebo		
	Aged 7–15	Aged 7–12	Aged 13–15	Aged 7–15	Aged 7–12	Aged 13–15
**ABC-T**						
Sensory	1.0	0.88	0.37	0.18	0.18	N.C.
Relating	1.0	0.72	0.21	0.84	1	0.39
Body and object use	0.04 *	0.06	0.48	0.5	0.5	N.C.
Language	0.33	0.48	0.37	0.31	0.39	0.39
Social and self help	0.4	0.46	0.62	0.78	0.78	N.C.
Total score	0.28	0.4	0.28	0.43	0.49	0.23
**SRS**						
Social communication	0.12	0.28	0.28	0.25	0.51	0.36
Autism mannerisms	0.08	0.21	0.13	0.19	0.19	0.89
Social awareness	0.93	0.9	0.7	0.41	0.62	0.52
Social emotion	0.09	0.33	0.08	0.29	0.38	0.55
Total score	0.04*	0.13	0.15	0.2	0.36	0.4
**CBCL**						
Anxiety	0.02 *	0.01 *	0.82	0.38	0.45	0.6
Withdrawn	0.43	0.45	0.85	0.63	0.63	1
Somatic complaints	0.85	0.9	0.7	0.1	0.16	0.39
Internalization	0.12	0.12	0.8	0.15	0.2	0.39
Social problems	0.21	0.32	0.3	0.66	0.93	0.18
Thoughts problems	0.05	0.06	0.62	0.17	0.22	0.53
Attention problems	0.46	0.24	0.46	0.78	0.7	0.79
Rule-breaking behavior	0.02 *	0.01 *	1	0.11	0.14	0.39
Aggressive behavior	0.41	0.42	0.85	0.07	0.13	0.31
External	0.11	0.1	1	0.02 *	0.04 *	0.32
Total score	0.1	0.09	0.83	0.3	0.48	0.4
**SNAP-IV**						
Inattention	0.08	0.03 *	0.49	0.91	0.86	0.72
Hyperactivity/impulsivity	0.04 *	0.02 *	0.59	0.32	0.29	0.72
Opposition/defiance	0.05	0.02 *	0.78	0.77	0.79	0.33
Total score	0.02 *	0.004 *	0.61	0.86	0.96	0.46

Data expressed are *P*-value. N.C.: difference equal to 0, the *p*-value is not calculable. * *P* < 0.05. ABC-T: Aberrant Behavior Checklist-Taiwan version; CBCL: Child Behavior Checklist; CGI-I: Clinical Global Impression-Improvement; CGI-S: Clinical Global Impression-Severity; SNAP-IV: Swanson, Nolan and Pelham (SNAP)-IV-Taiwan version; SRS: Social Responsive.

**Table 4:** Age stratified analysis of outcomes for subjects aged 7–12 years old.

	Baseline			Week 4			Difference between PS128 and placebo
	PS128	Placebo	*P*-Value	PS128	Placebo	*P*-Value	*P*-Value
**CGI-I**				3.68 (1.14)	3.57 (1.04)	0.69	
**ABC-T**							
Sensory	2.36 (1.47)	2.9 (1.88)	0.22	2.29 (1.61)	2.68 (1.68)	0.38	0.41
Relating	4.14 (2.89)	3.66 (2.35)	0.49	4.43 (3.1)	3.77 (2.72)	0.41	0.82
Body and object use	3.32 (2.75)	3.7 (2.74)	0.6	3.04 (2.67)	3.52 (2.75)	0.51	0.51
Language	2.44 (2.26)	3.1 (1.81)	0.23	2.14 (1.94)	2.93 (1.84)	0.13	0.93
Social and self help	3.28 (2.12)	3.45 (2.06)	0.75	3.21 (2.08)	3.41 (2.44)	0.75	0.71
Total score	16.22 (8.79)	16.82 (9.08)	0.8	15.11 (9.14)	16.36 (10.16)	0.64	0.95
**SRS**							
Social communication	63.6 (15.54)	64.5 (16.5)	0.83	62.12 (12.89)	64.79 (16.01)	0.51	0.72
Autism mannerisms	30.53 (6.59)	31.1 (6.77)	0.75	30.04 (7.52)	30.33 (6.47)	0.88	0.82
Social awareness	23.59 (4.42)	20.73 (4.73)	0.02 *	23.46 (5.29)	20.62 (4.67)	0.04 *	0.74
Social emotion	19.41 (4.66)	19.53 (4.21)	0.92	19.25 (4.66)	19.25 (4.88)	1	0.98
Total score	138.23 (25.67)	137.25 (27.28)	0.89	132.72 (23.37)	137.96 (25.98)	0.46	0.43
**CBCL**							
Anxiety	6.86 (5.2)	6.14 (4.43)	0.86	5.59 (4.34)	5.82 (4.39)	0.83	0.13
Withdrawn	4.45 (3.03)	4.59 (2.95)	0.35	4.26 (2.7)	4.43 (3.02)	0.71	0.81
Somatic complaints	2.44 (1.91)	3.08 (2.88)	0.98	2.63 (2.63)	2.36 (2.51)	0.97	0.65
Internalization	13.5 (8.63)	13.56 (8.13)	0.81	12.48 (6.9)	12.56 (7.87)	0.95	0.78
Social problems	7.83 (4.24)	7.59 (3.29)	0.32	7.7 (4.07)	7.63 (3.81)	0.2	0.44
Thoughts problems	5.96 (4.22)	7.37 (5.79)	0.59	5.08 (4.37)	6.81 (5.39)	0.95	0.41
Attention problems	10.54 (3.72)	11.07 (3.73)	0.77	11 (4.2)	10.93 (4.18)	0.98	0.25
Rule-breaking behavior	3.72 (3.38)	3.96 (2.79)	0.87	3.23 (3.65)	3.25 (3.31)	0.81	0.39
Aggressive behavior	8.46 (6.86)	8.76 (6.1)	0.73	8.22 (6.44)	7.82 (6.09)	0.89	0.87
External	12.08 (9.75)	12.96 (8.56)	0.89	11.42 (9.6)	11.07 (8.67)	0.45	0.83
Total score	51.04 (26.87)	52.14 (26.9)	0.86	45.17 (23.02)	50.5 (24.42)	0.83	0.29
**SNAP-IV**							
Inattention	15.29 (5.58)	15.43 (5.14)	0.92	14.18 (5.72)	15.26 (5.65)	0.48	0.08
Hyperactivity/impulsivity	10.75 (5.75)	11.41 (5.96)	0.67	9.88 (5.22)	10.82 (6.5)	0.56	0.26
Opposition/defiance	9.26 (5.93)	7.38 (5.13)	0.21	7.71 (4.75)	7.32 (5.44)	0.77	0.03 *
Total score	34.85 (14.46)	34.5 (13.8)	0.93	31.88 (13.83)	33.59 (15.99)	0.68	0.02 *

The results are expressed as means (SD). * *P* < 0.05. ABC-T: Aberrant Behavior Checklist-Taiwan version; CBCL: Child Behavior Checklist; CGI-I: Clinical Global Impression-Improvement; CGI-S: Clinical Global Impression-Severity; SNAP-IV: Swanson, Nolan and Pelham (SNAP)-IV-Taiwan version; SRS: Social Responsiveness Scale.

## Supplementary Materials

The following are available online at https://www.mdpi.com/2072-6643/11/4/820/s1, Figure S1: Numbers of patients diagnosed with autism spectrum disorder in Taiwan in the recent 10 years.
