# Supplementary material for: Effects of Lactobacillus plantarum PS128 on Children with Autism Spectrum Disorder in Taiwan: A Randomized, Double-Blind, Placebo-Controlled Trial
Source: Nutrients. 2019 Apr 11;11(4):820. doi: 10.3390/nu11040820 (PMC6521002; doi:10.3390/nu11040820)
Supplement: Supplementary file 1 [file nutrients-11-00820-s001.pdf]

Supplementary Materials

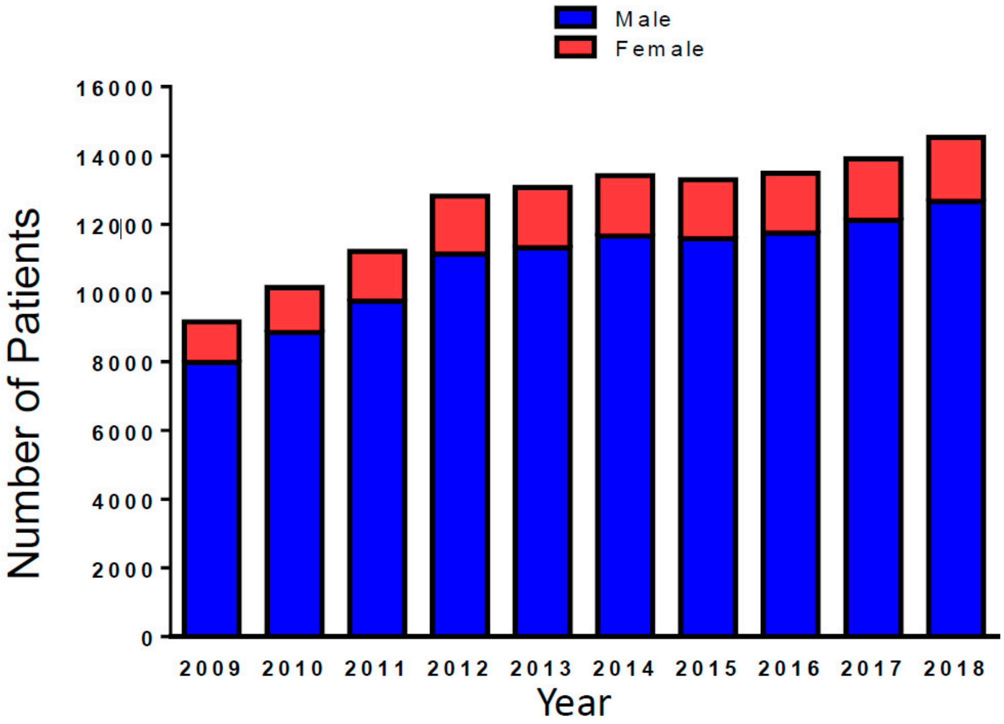

Figure S1. Numbers of patients diagnosed with autism spectrum disorder in Taiwan in the recent 10 years.
